# Hydrothermal synthesis of hydroxyapatite powders using Response Surface Methodology (RSM)

**DOI:** 10.1371/journal.pone.0251009

**Published:** 2021-05-20

**Authors:** Shamsi Ebrahimi, Coswald Stephen Sipaut@ Mohd Nasri, Sazmal Effendi Bin Arshad

**Affiliations:** 1 Chemical Engineering, Faculty of Engineering Universiti Malaysia Sabah, UMS, Sabah, Malaysia; 2 Biotechnology, Faculty of Sciences and Natural Resources, Universiti Malaysia Sabah, UMS, Sabah, Malaysia; Texas A&M University at Qatar, QATAR

## Abstract

Hydroxyapatite (HAp)—[Ca_10_ (PO_4_)_6_(OH) _2_] has a similar chemical composition to bone material, making it the main mineral supplement in bone-making. Due to its high biocompatibility, hydroxyapatite is widely used in the repair of bone deficiencies and in the production of dental or orthopedic implants. In this research, hydroxyapatite nanopowder was synthesized using a hydrothermal technique. Fourier Transform Infrared Spectroscopy (FTIR) and transmission electron microscopy (TEM) were used to investigate the chemical structure and morphology of the synthesized hydroxyapatite powder. X-ray diffraction (XRD) was used to evaluate the phase analysis of HAp nanopowder. In addition, bioactivity HAp assessment was conducted by scanning electron microscopy (SEM) attached with Energy Dispersive X-Ray Spectroscopy (EDX) analysis. Response Surface Methodology (RSM) with central composite design (CCD) was used in order to determine the optimal conditions for yield, size, and crystallinity. Three independent variables (pH, temperature, and hydrothermal treatment time) were investigated. The yield was observed to increase in alkaline conditions; pH showed the greatest influence on the yield, size, and crystallinity of the synthesized hydroxyapatite, based on Analysis of Variance. The results of bioactivity evaluation are showed high bioactivity due to the formation of apatite on the surface of the synthesized nanopowder.

## Introduction

Calcium phosphate is one of the main mineral components of the skeleton, making it an ideal material for bone grafts in bone tissue engineering [1]. Due to the high biocompatibility and biological activity of calcium phosphate, applications to nanomaterial structures are currently widely investigated [2, 3]. Recently, hydroxyapatite (HAp) has been used in the medical sciences as an important and widely applicable bioceramic, similar to the mineral phase of human hard tissue. This similarity between the chemical composite of hydroxyapatite and bone is the main factor in its bioactivity, its ability to stimulate bone growth, and its therapeutic effects [4, 5]. Hydroxyapatite has been widely synthesized at a nanoscale. Various methods have been used to produce hydroxyapatite nanocrystals, which, depending on the method, generate various products with different structures, apparent features, and crystalline rates. The main HAp synthesis techniques are wet chemical methods and dry reactions [6–11].

Dry reaction methods include solid-state reactions and mechanochemical methods, whereas wet reaction methods include chemical sedimentation, hydrolysis reactions, sol-gel, hydrothermal, emulation, and sonochemical methods. Wet methods allow the size and morphology of nanoparticles to be controlled and are therefore promising techniques for nanoparticle production [12]. The main advantage of ceramic materials synthesized using hydrothermal methods compared to others, such as sol-gel synthesis, is that relatively low temperatures are required to produce the ceramic body [13]. The hydrothermal process is a wet chemical technique able to direct the formation of complex oxide powders with high crystallinity, making it the most popular method used to prepare hydroxyapatite nanoparticles. The advantage of this method, especially in the synthesis of hydroxyapatite powders, is that no hydroxyl defects are produced in the structure. Hydrothermal processes usually work with a chemical reaction in an aqueous solution at high temperature and pressure. Due to its high biocompatibility, this has become a favored technique for material production [14]. Several parameters determine the synthesis of hydroxyapatite nanoparticles. Multiple tests and considerable time are required to determine the optimal conditions of synthesis [15]. Parameters such as temperature, the time of hydrothermal reaction, and the pH of the solution play a significant role in the synthesis of hydroxyapatite nanoparticles with desired properties. Experimental statistical methods such as Response Surface Methodology (RSM) can be used to investigate the effect of these parameters (i.e. pH, hydrothermal reaction time, and temperature). Response surface methods are a set of statistical and mathematical methods that may be used for optimization and modelling and can also evaluate the interactions of multiple variables [15]. In this research, the RSM method with Central Composite Design (CCD) was used to find out the most influential process parameters on the reaction yield, nanoparticle size, and degree of crystallinity of prepared hydroxyapatite nanoparticles. The parameters studied were pH, reaction temperature, and the time of hydrothermal treatment.

## Materials and methods

### Materials

The raw materials used in this work included calcium nitrate tetrahydrate (Ca (NO_3_)_2_.4H_2_O) and diammonium hydrogen phosphate ((NH_4_)_2_HPO_4_). Ammonia solution (NH_3_) and nitric acid (HNO_3_) for adjusting pH were also used. All chemicals were obtained from Merck.

### Experimental design

The effects of pH, hydrothermal temperature, and time on the obtained hydroxyapatite were investigated via Response Surface Methodology (RSM) with Central Composite Design (CCD). Each parameter was investigated at five levels (−α, −1, 0, +1, +α), as shown in Table 1, pH (1, 4, 7, 10, 13) hydrothermal temperature (70, 100, 130, 160, 190°C) and hydrothermal reaction time (1, 4, 7, 10, 13 h). In the design of experiments using CCD, the number of experiments is determined by the following Eq.:

$$\text{Y}=2^{\text{x}}+2\text{x}+{\text{C}}_{\text{x}}$$ (1)

Y is the number of experiments, x is the number of variables, and Cx is the number of center points repetitions [15]. In this study, the number of variables is 3, and the number of center points and repetitions are 3 and 1, respectively. A total of 17 runs were generated by this design.

**Table 1:** Experimental parameters and their levels.

Sample	parameter	(+1)	(0)	(-1)	-α	+α
A	Temperature (°C)	100	130	160	70	190
B	Time (h)	4	7	10	1	13
C	pH	4	7	10	1	13

In Table 1, alpha (α) = 2 (star or axial point for orthogonal CCD in the case of three independent variables), and the values are shown are rounded. The mathematical relationship between the independent and response variables was modeled using the following second-degree polynomial Eq.:

$$\text{y}={\text{b}}_{0}+\sum_{\text{i}=1}^{\text{n}}{\text{b}}_{\text{i}}{\text{x}}_{\text{i}}+\sum_{\text{i}=1}^{\text{n}}{\text{b}}_{\text{ii}}{\text{x}}_{\text{i}}^{2}+\sum_{\text{i}=1}^{\text{n}}\sum_{\text{j}>1}^{\text{n}}{\text{b}}_{\text{ij}}{\text{x}}_{\text{i}}{\text{x}}_{\text{j}}$$ (2)

Where b_0_ is a constant, b_i_ are linear coefficients, and x_i_ is the effect of a single variable. The effect of interaction parameters are x_i_x_j_. b_ij_ is the interaction coefficient, and b_ii_ is the coefficient representing the effect of interaction between variables x_i_x_i_ [16].

Eq 3 below was used to calculate the reaction efficiency in different conditions based on the RSM method [17]. In order to obtain the pure powder, the water used as a reaction environment was exposed to 60°C heat for 10 hours to evaporate completely. In Eq 3, w_1_ is the weight of powder before drying, and w_2_ is the weight of powder after drying.

$$\text{Yield}\;\%=\frac{W_{2}}{W_{1}}\times 100$$ (3)

### Sample preparation

0.67 M ammonium hydrogen phosphate solution was added dropwise to a solution of 1 M of calcium nitrate tetrahydrate under an appropriate stirring for 1 hour (stoichiometric ratio ca / p = 1.67 [18]).,The pH of reactants was adjusted to the range 1 to 13 using ammonia solution and nitric acid. The hydrothermal synthesis involved transferring hydroxyapatite suspension to a Teflon vessel 150 ml and allowing reactions to proceed at different temperatures and times, as shown in Table 1. Finally, the white suspension was filtered, and the hydroxyapatite powder was washed three times with deionized water and ethanol (volume ratio of 1:1) to remove impurities. The powder was dried at 60°C for 10 hours and crushed with a mortar and pestle.

### Preparation of simulated body fluid (SBF)

The SBF solution was prepared based on the Kokubo guidelines. The quantity of sodium chloride salts, sodium hydrogen carbonate, potassium chloride, dipotassium hydrogen phosphate triplets, and magnesium chloride 6 hydrate were calculated and prepared as in Table 2. The reagents were then dissolved one by one in 1 L distilled water, and the solution was buffered to pH = 7 with a solution of tris-hydroxyethyl aminomethane and hydrochloric acid.

**Table 2:** Ion concentrations of prepared SBF (Simulated Body Fluid) and human blood plasma.

Ion	Concentration (m Mol.dm-3)	
	(SBF)	Human blood plasma
Na^+^	142.0	142.0
K^+^	5.0	5.0
Mg^2+^	1.5	1.5
Ca^2+^	2.5	2.5
Cl^-^	147.8	103.0
HCO^-3^	4.2	27.0
HPO_4_^-2^	1.0	1.0
SO_4_^2-^	0.5	0.5

### Characterization

The identification of functional groups in the synthesised HAp samples was conducted using FT-IR spectroscopy. The FTIR spectra were recorded using Perkin- Elmer spectrum 100 FTIR spectrophotometer in the range of 400–4000 cm^-1^. The SEM (Hitachi S3400 N), equipped with EDX (Quantex200), was used to study the samples morphology and chemical composition analysis. Transmission electron microscopy (Model FEL, Tecnai Spirit BioTWIIN) was utilized to examine the morphology and estimate the sample particle size. XRD was carried out using an X-ray diffractometer (X’pert Pro, Philips) with a CuKa radiation source (λ = 1.54056 Å) and operated at 40 kV and 30 mA. Diffraction patterns at room temperature were recorded over the 2θ range 10–80°, with a step size of 0.02° and time per scan of 1 sec. After obtaining XRD patterns, each of the phases was determined by comparing the angle and intensity of diffraction peaks with information on the following ICCD standard cards: HAp (PDF Card no.9-432), monotite (DCPA, CaHPO_4_, PDF Card no. 09–0080), octacalcium phosphate (OCP, C_8_(HPO_4_)_2_(PO_4_)_4_.5H_2_O, PDF Card no. 26–1056), which is in accordance with hexagonal crystal form. In order to obtain the size of the synthesized HAp crystals, the peak of the L_002_ plane of HAp was taken to be 2θ = 25.80–26.6°. The diffraction peak width of the plane (002) was measured at half of the maximum intensity. Then, crystal size was calculated using the Scherer relation (Eq 4), and the degree of crystallinity was measured using Eq 5 [19, 20].

$$\text{D}=\frac{\text{K}\lambda }{\beta \text{cos}\theta }$$ (4)

$${\text{X}}_{\text{c}}(\text{\%})=\frac{\sum {\text{A}}_{\text{c}}}{\sum {\text{A}}_{\text{c}}+\sum {\text{A}}_{\text{A}}}\times 100$$ (5)

Where D is the crystal size, *β* is the FWHM (full width at half maximum (in radians)) of the (002) reflection, Ѳ is the diffraction angle (ᵒ), K is the shape factor (equal to 0.9), and λ (λ = 1.5405 Ǻ) is the X-ray wavelength [21]. ΣA_C_ + ΣA_A_ gives the sum of the area under all the HAp crystalline and amorphous peaks, and ΣA_C_ yields the sum of the areas under the crystalline peaks present in the scan range between 10° to 80°.

## Results and discussion

**Fig 1:**
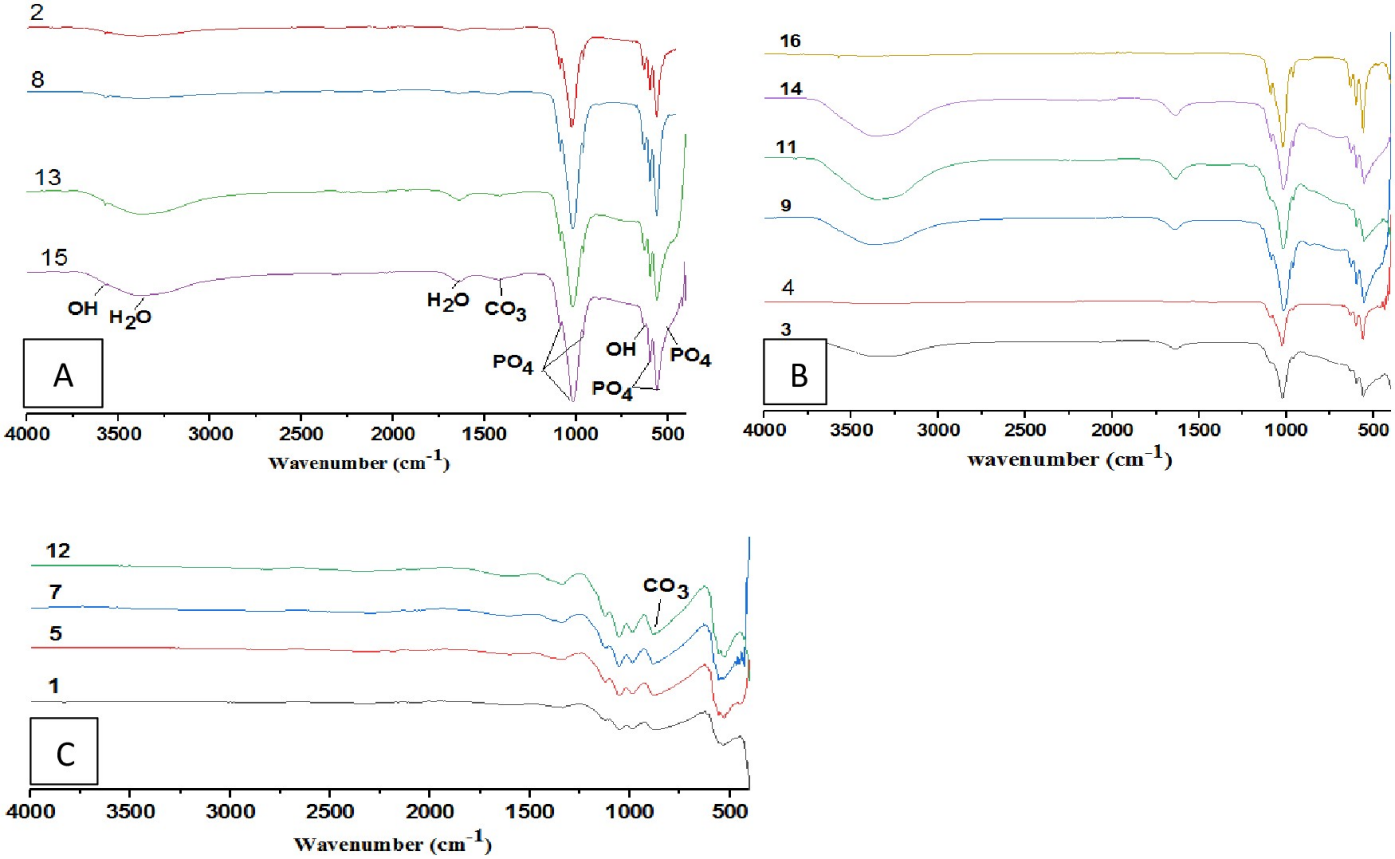
FTIR spectra of HAp powders at different condition based on RSM design. A) Alkaline condition; B) Normal condition; C) Acidic condition.

The FTIR results are shown in Fig 1. From Fig 1A and 1B, the bands at 465, 559.51, and 598.92 cm^-1^ are attributed to the bending vibration of phosphate (PO_4_^-3^), and the bands appearing at 962.18, 1022.58, and 1088.45 cm^-1^ are related to its stretching vibration. Broad peaks are observed between 3300–3450 cm^-1^ and at 1367 cm^-1^, showing the bending of water hydroxyl (OH^-^). Since the hydroxyapatite was synthesised in an aqueous medium, this might be from the absorption of water into its structure. The two weak peaks at 3570 cm^-1^ and 631.70 cm^-1^ correspond to the stretching of OH^-^ ions in the HAp structure [22], suggesting that all functional groups of (OH^-^, PO_4_^-3^) appeared in alkaline conditions (Fig 1A). At a low pH (Fig 1C), the presence of hydroxyl groups in the hydroxyapatite lattice was diminished, resulting in the formation of calcium carbonates. This is shown by the presence of peaks between 1420–1450 cm^-1^ and at 864 cm^-1^, which can be attributed to carbonate CO_3_ functionality [23]. A carbonate (CO_3_^-2^) peak was seen in alkaline conditions at 1430 cm^-1^, formed from both CO_2_ in the atmosphere and sufficient hydroxyl in the alkaline conditions.

The crystallinity profile was also determined from the FTIR spectra. The sharpness and intensity of hydroxyapatite groups (hydroxyl group at 3570 cm^-1^, phosphate group at 962.18 cm^-1^) can indicate hydroxyapatite crystallinity [24]. Therefore, with the increase in temperature from 130°C to 190°C, the hydroxyl group clearly corresponded to hydroxyapatite. At 190°C, the functional groups of hydroxyapatite are quite sharp (refer to sample 16). With increased hydrothermal reaction time, the intensity and sharpness of the peaks associated with hydroxyapatite also increase [24–26].

**Fig 2:**
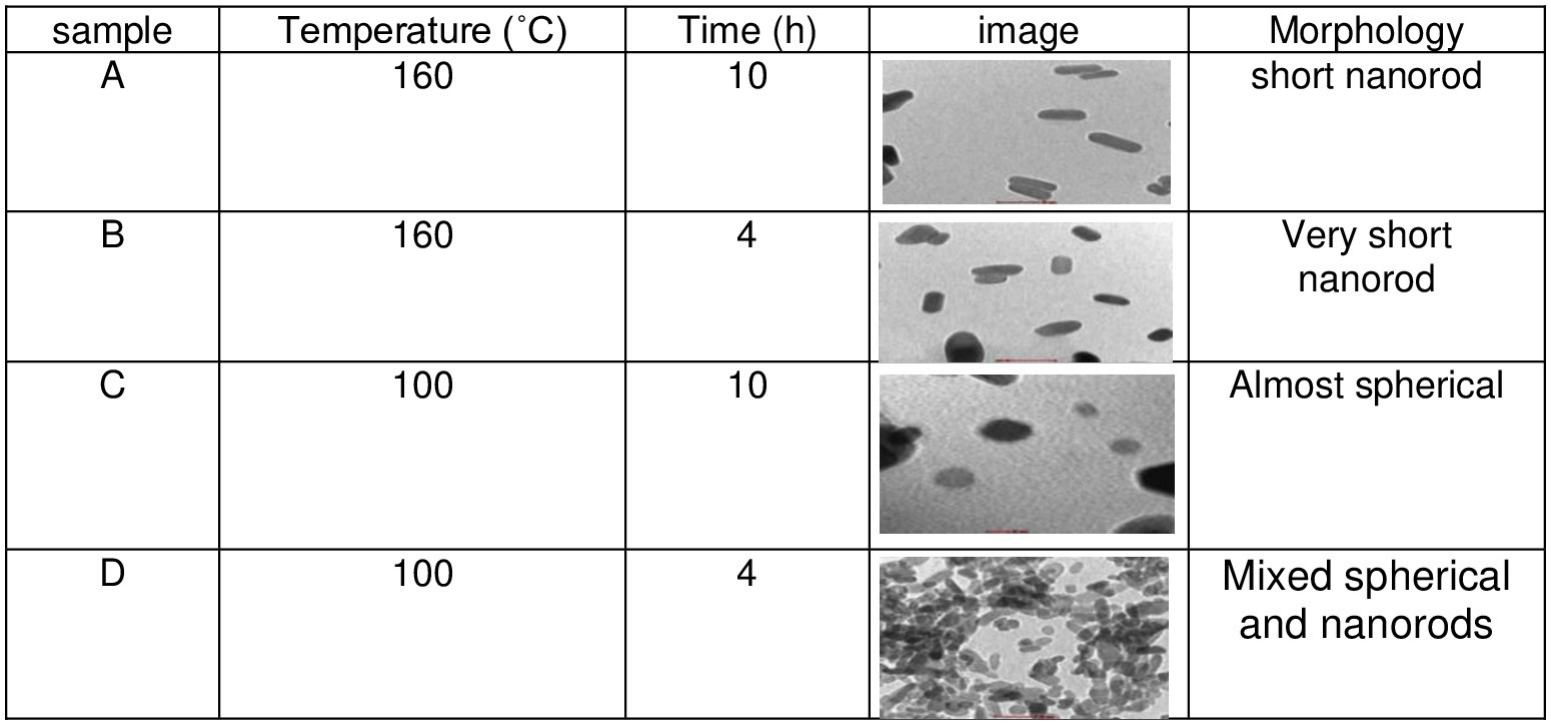
TEM images for HAp samples in alkaline conditions (pH = 10).

**Fig 3:**
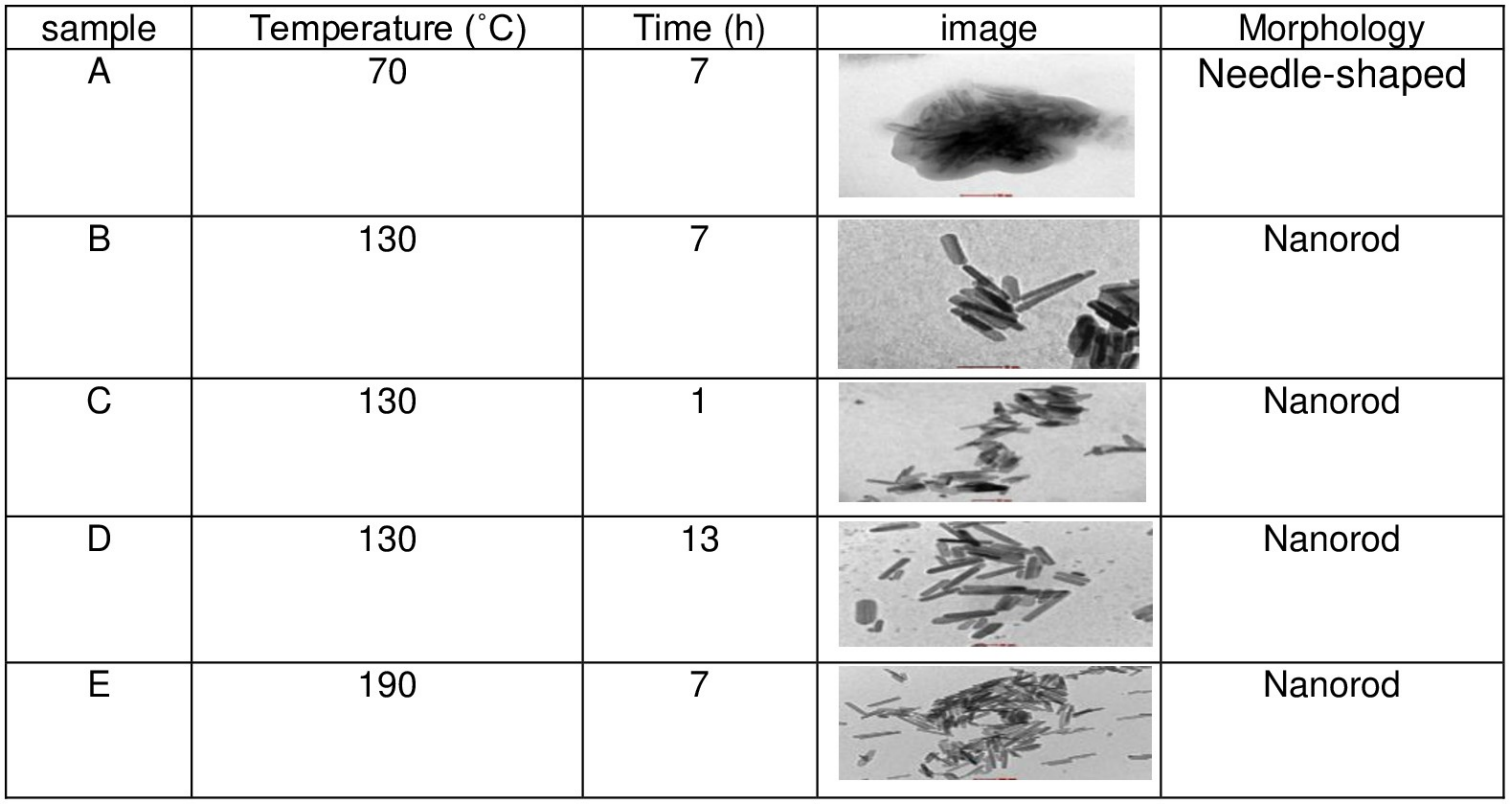
TEM images for HAp samples in normal conditions (pH = 7).

**Fig 4:**
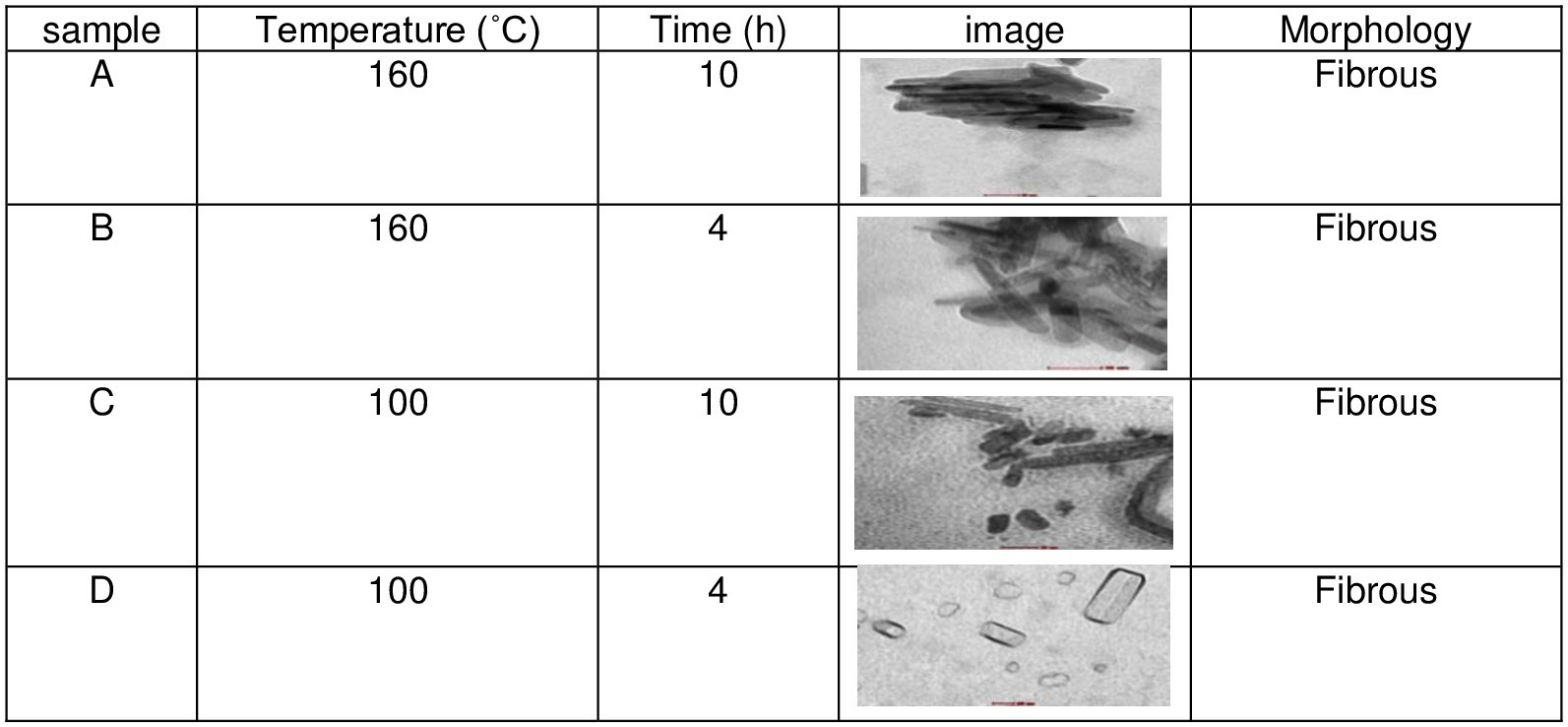
TEM images for HAp samples in acidic conditions (pH = 4).

The TEM results are shown in Figs 2–4 and illustrate the morphology and the size of the hydroxyapatite nanopowder at three different pH levels: alkaline, normal, and acidic (pH = 10, 7, and 4, respectively). Fig 2 shows that the particles under alkaline conditions are close to spherical in shape and have diameters in the range of 20 to 50 nm. In alkaline conditions and with hydrothermal reaction time fixed at 4 h, the particles increased in size with temperature (100°C– 160°C), suggesting that particle morphology depends greatly on the temperature of the hydrothermal process. Most previous research shows that an increase in hydrothermal process temperature results in nanoparticles with increased length but decreased diameter and that particles grow along the C-axis [27]. Fig 2 also shows that particles are smaller and less elongated in alkaline conditions, with convergent growth [24]. In alkaline conditions, nanoparticles grow isotropically, resulting in spherical crystal growth [12]. With high pH and low temperature, convergent growth occurs when strong ions of OH^-^ are attracted onto calcium phosphate, making the HAp particles spherical [28]. Using a hydrothermal method, rod-shaped and needle-like nanoparticles are obtained under normal conditions (Fig 3) and acid conditions (Fig 4), respectively [12].

Several studies have been conducted on the synthesis of hydroxyapatite nanoparticles in acidic conditions. These studies show that the resulting nanoparticles are rod-shaped. By increasing the hydrothermal time (4–10 h) at a fixed temperature (160°C), the length of HAp nano bars increases linearly, whereas diameter reduces. Therefore, thinner and longer nanoparticles can be obtained by increasing the hydrothermal reaction time [27].

### Investigating phase changes using XRD

**Fig 5:**
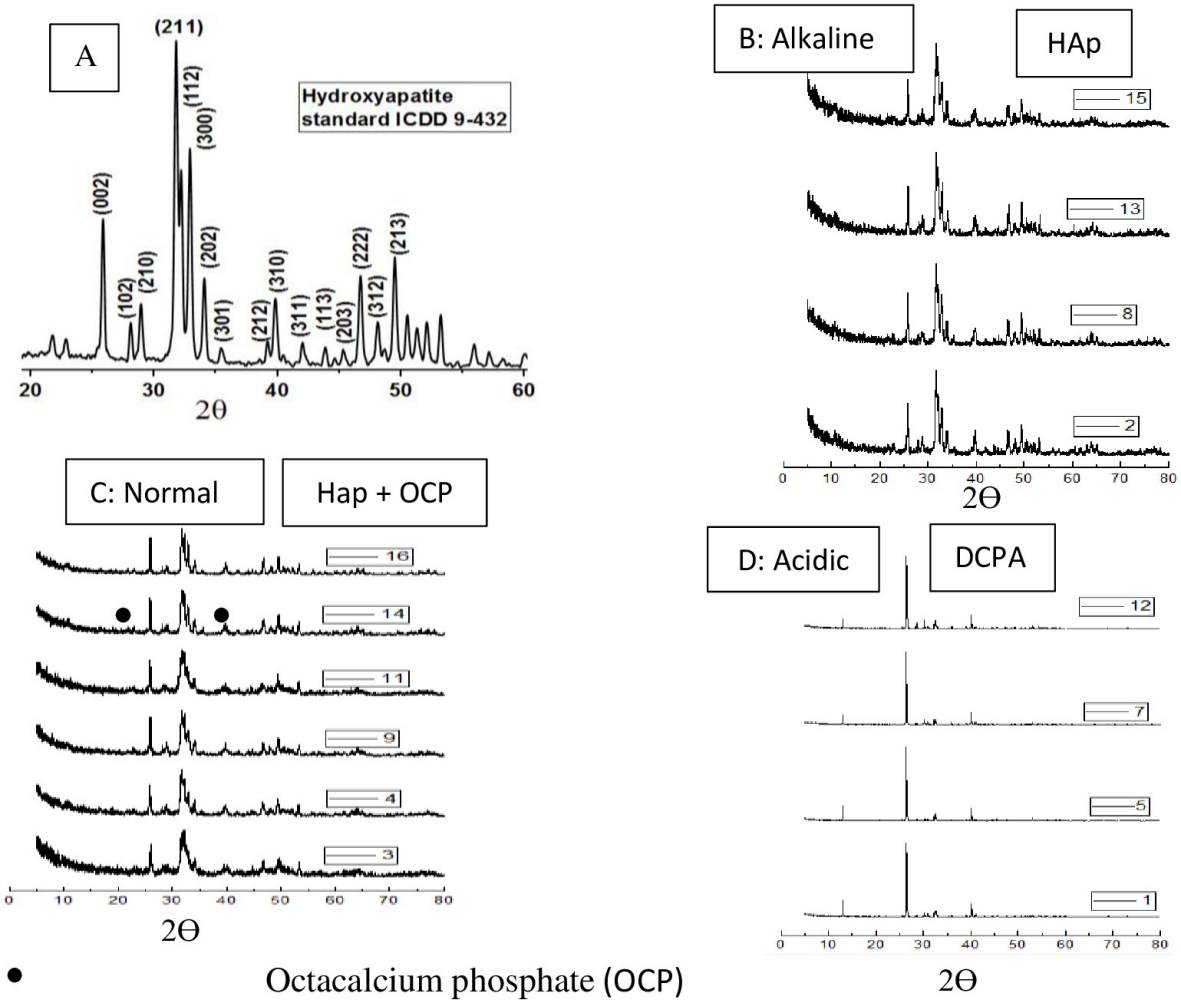
XRD pattern hydroxyapatite standard [29] and XRD pattern at different condition based on RSM design.

XRD was used to obtain the size of the synthesized HAp crystals. Fig 5 shows XRD patterns for all 17 HAp samples under different conditions of pH, temperature, and hydrothermal reaction time. The peak of the L_002_ plane of HAp was selected as a base peak (2θ = 25.80–26.6°) in order to measure crystal size. This selected peak was attributed to the first peak of HAp due to its sharpness and distance from other peaks [19]. It is noted that sample 6 produced no powder due to the dissolution of the sample at pH < 2. As demonstrated by the XRD patterns in Fig 5B, pure HAp is obtained in alkaline conditions.

In the acidic conditions shown in Fig 5D, HAp is unstable, and at pH = 4, calcium phosphates with calcium deficiency are formed. In sample 1, where temperature = 100°C, time = 4 h, and pH = 4, only the monotite phase (DCPA, CaHPO_4_) was formed. These results are in agreement with earlier findings [12]. In reaction 5 where pH = 4, only the monotite phase was formed. Peaks are sharper in reaction 5 due to higher temperature = 160°C.

Under normal conditions (Fig 5C and labeled as 3, 4, 9, 11, 14, 16), calcium hydrogen phosphate hydrate (OCP (C_8_ (HPO_4_)_2_(PO_4_)_4_.5H_2_O) phase is also formed in addition to the HAp phase.

Table 3 shows the crystal size, percentage of crystallinity, and the yield of the produced products at different conditions. From Table 3, it can be observed that at low temperatures, the percentage of crystallinity also is low. This behavior is shown for sample 3. Further, by increasing hydrothermal temperature, the octacalcium phosphate (OCP) phase decreases, and the HAp phase growth increases, as shown in sample 3 and sample 16. Results have also shown that increasing reaction time from 4 to 10 hours, the size of nanoparticle produced increases and the crystallinity increases. This behavior can identify in sample 8 and sample 2.

**Table 3:** XRD results for 17 sample HAp.

Run	A	B	C	Responses	
	Temperature (°C)	Time (h)	pH	Crystal size	Crystallinity (%)
1	100	4	4	60.23	55.83
2	160	10	10	45.48	86.13
3	70	7	7	28.76	68.86
4	130	7	13	46.81	72.92
5	160	4	4	84.10	58.07
6	130	7	1	-	-
7	100	10	4	65.15	57.50
8	160	4	10	38.98	82.53
9	130	7	7	45.82	76.85
10	130	7	7	46.88	77.89
11	130	1	7	44.10	75.16
12	160	10	4	89.00	59.14
13	100	10	10	31.59	78.88
14	130	13	7	56.18	80.01
15	100	4	10	29.18	74.82
16	190	7	7	71.15	79.84
17	130	7	7	44.77	74.82

The particle morphology and crystallinity of crystals are strongly influenced by the hydrothermal temperature [27]. With an increase in temperature, XRD peaks become sharper, the degree of crystallinity increases, and the amorphous phases change to crystal phase, with an increased sedimentation rate [27]. From the XRD patterns, the minimum temperature required for the synthesis of HAp is 100°C, and higher temperatures are desirable for greater crystallinity. At the lower temperature of 70°C (Table 3, sample 3), compounds are produced with calcium deficiency, whereas at higher temperatures, these amorphous phases change to HAp crystals [12].

Crystal size increases in acidic conditions because the crystals tend to grow along the C-axis. Crystal growth rises with increased reaction time since the increase of hydrothermal process time promotes solvent evaporation and oversaturates the remaining solution, leading to crystal growth [25]. From Table 3, it can be seen that crystal size also increases with hydrothermal temperature, which is in accordance with the results of other research [27].

### Experimental analysis using the RSM method

A total of 17 experiments were carried out and compared to the results obtained from Design Expert software (version 11). The parameter values for each experiment (actual data) and from Design Expert (predicted data) are shown in Table 4.

**Table 4:** Design of Central Composite Design (CCD) and its actual values.

Run	A	B	C	Yield (%)		Crystal size (nm)		Crystallinity (%)	
	Temp* (°C)	Time (h)	pH	Actual	Predict	Actual	Predict	Actual	**Predict**
1	100	4	4	46.18	43.33	60.23	59.59	55.83	55.88
2	160	10	10	89.75	92.79	45.48	47.00	86.13	86.82
3	70	7	7	65.44	72.03	28.76	28.00	68.86	68.88
4	130	7	13	84.61	84.88	46.81	45.14	72.92	72.18
5	160	4	4	48.70	46.83	84.10	85.12	58.07	58.48
6	130	7	1	0	-	-	-	-	
7	100	10	4	47.5	50.04	65.15	64.95	57.50	57.43
8	160	4	10	83.80	86.08	38.98	41.63	82.53	83.34
9	130	7	7	74.74	75.53	45.82	47.52	76.85	77.88
10	130	7	7	75.85	75.53	46.88	47.52	77.89	77.88
11	130	1	7	68.25	68.82	44.10	44.16	75.16	74.70
12	160	10	4	51.64	53.54	89.00	90.48	59.14	59.49
13	100	10	10	91.00	89.29	31.59	33.48	78.88	79.21
14	130	13	7	88	82.25	56.18	52.88	80.01	79.72
15	100	4	10	87	82.58	29.18	28.12	74.82	75.21
16	190	7	7	78.34	79.04	71.15	67.04	79.84	79.08
17	130	7	7	77.3	75.53	44.77	47.52	74.82	75.21

*Temperature

### Analysis of result variation for reaction yield

The first level of result analysis is the selection of a suitable model for the system to predict the results accurately. For this reason, the suggested second grade polynomial and second grade quadratic models were used. Usually, the ANOVA variance (Table 5) is used for the evaluation of the model and testing its meaningfulness. The result of reaction yield is indicated in Table 5. Table 5 also indicates the importance of model adequacy scales such as correlation coefficient (R^2^ = 0.97), (R^2^adj = 0.948), (predicted R^2^ = 0.80), and (adequate precision = 17.28). Adequate precision with value (17.28) indicates the noise signal. In general, an adequate precision value higher than 4 is suitable. Hence, the ratio obtained in this model is adequate (17.28) [30].

**Table 5:** ANOVA table for yield (quadratic model).

Source	Sum of Squares	df	Mean square	F Value	P-value Prop>F	
Model	3746.46	9	416.27	31.78	0.0002	Significant
A-Temp	49.04	1	49.04	3.74	0.1011	
B-Time	180.30	1	180.30	13.77	0.0100	
C-pH	3431.24	1	3431.24	261.99	< 0.0001	
AB	1.59	1	1.59	0.1216	0.7392	
AC	15.43	1	15.43	1.18	0.3194	
BC	4.05	1	4.05	0.3090	0.5984	
A^2^	19.52	1	19.52	1.49	0.2680	
B2	5.93	1	5.93	0.4531	0.5259	
C2	593.79	1	593.79	45.34	0.0005	
Residual	78.58	6	13.10			
Lack of Fit	75.28	4	18.82	11.42	0.0821	Not significant
Pure Error	3.30	2	1.65			
Corrected Total	3825.04	15				

R^2^ = 0.97, Predicted R^2^ = 0.80, adjusted R^2^ = 0.948, adequate precision = 17.28

Reaction yield based on coding factors is shown in Eq 6.

$$\begin{array}{l} \text{Yied}=+76.07+1.75*\text{A}+3.36*\text{B}+19.61*\text{C}+0.4463*\text{A}*\text{B}-1.39*\text{A}*\text{C}+\\ 0.7112*\text{B}*\text{C}-1*{\text{A}}^{2}+0.5540*{\text{B}}^{2}-7.59*{\text{C}}^{2} \end{array}$$ (6)

Reaction yield based on real factors is shown in Eq 7.

$$\begin{array}{l} \text{Yield}=\\ -47.925+0.421*\text{Temp}-0.940*\text{Time}+17.793*\text{pH}+0.00495*\text{Temp}*\text{Time}-\\ 0.01543*\text{Temp}*\text{pH}+0.079028*\text{Time}*\text{pH}-\\ 0.0011*{\text{Temp}}^{\text{2}}+0.0615*{\text{Time}}^{\text{2}}-0.843*{\text{pH}}^{\text{2}} \end{array}$$ (7)

By retaining only factors with p ≤ 0.05, a final model for predicting reaction yield according to the remaining system parameters was derived (Eq 8).

$$\text{Yield}=-26.365+0.0583*\text{Temp}+1.118*\text{Time}+18.167*\text{pH-}\;0.830*{\text{pH}}^{\text{2}}$$ (8)

### Influence of effective parameters on reaction yield

Eq 6 was used to calculate the reaction yield under different conditions based on the RSM method [17].

### Effect of hydrothermal temperature on reaction yield

**Fig 6:**
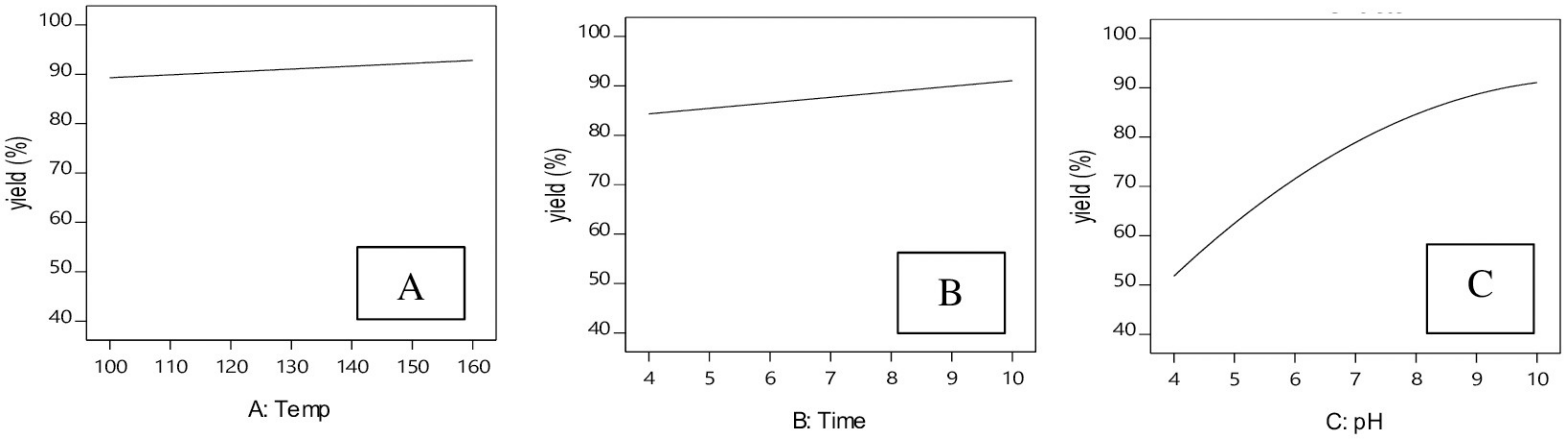
Effect of process parameters on reaction yield. A: Hydrothermal temperature, B: hydrothermal reaction time, C: pH.

Fig 6A shows the effect of temperature on reaction yield. It is seen that as temperature increases, yield shows a first order increase with a slow ascending slope. Hence, reaction yield does not depend strongly on the hydrothermal reaction temperature.

### Effect of hydrothermal reaction time on reaction yield

Fig 6B shows the effect of hydrothermal reaction time on reaction yield. With increased hydrothermal process time, reaction yield also only increases with a very slow ascending slope, suggesting that reaction yield has no significant relationship with hydrothermal time.

### Effect of pH on reaction yield

Inspection of Eq 8 shows that pH exerts the greatest influence on reaction yield since it is a quadratic term (Fig 6C). From the curves in Fig 6C, increasing pH increases reaction yield quadratically with an ascending slope. In other words, the increased yield is directly related to increased pH. When pH = 4, reaction yield is about 50%, but when pH = 10, reaction yield reaches approximately 90%.

Based on the results of Table 4, the maximum reaction yield was produced in alkaline conditions, with the minimum produced in acidic conditions. This suggests that reaction yield is highly dependent on pH. Consistent with previous research [31], this study also indicates that HAp is stable in alkaline conditions and unstable at lower pH, with total dissolution occurring at pH ≤ 2.

**Fig 7:**
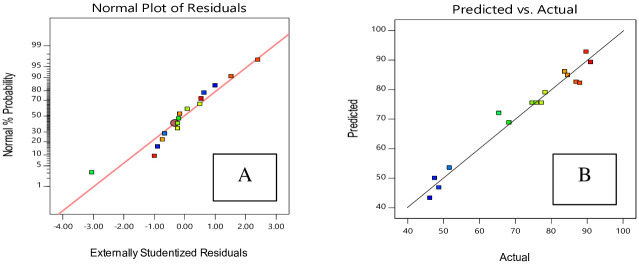
Residual plots for yield of HAp nano powder, based on Central Composite Design (CCD). A: normal probability; B: residuals.

A normal probability diagram indicates how closely residuals (differences between experimental and predicted values) follow a normal distribution (Fig 7A). Fig 7A shows that the data are well correlated. The relation between the real data and predicted data is indicated in Fig 7B. The predicted results and experimental results are highly similar and correlate strongly with each other [17].

### Variance analysis for the degree of crystallinity response

The variance analysis for the degree of crystallinity response indicated that a quadratic model is statistically recommended for more analysis, since it had the greatest R^2^ prediction and R^2^adj. A regression importance test for the model was carried out, and additional importance tests of individual model coefficients and lack of fit test were done. Table 6 shows the variance analysis of each response and the important terms in the reduced second degree model. Table 6 also presents model adequacy measures such as R^2^, R^2^adj, predicted R^2^, and adequate precision. The obtained adequate precision value of 35.64 indicates a noisy signal.

**Table 6:** ANOVA table for crystallinity (quadratic model).

Sorce	Sum of Squares	df	Mean square	F Value	P-value Prop>F	
Model	1443.45	9	160.38	133.10	< 0.0001	Significant
A-Temp	104.00	1	104.00	86.32	< 0.0001	
B-Time	25.22	1	25.22	20.93	0.0038	
C-pH	1213.74	1	1213.74	1007.30	< 0.0001	
AB	0.1418	1	0.1418	0.1177	0.7433	
AC	15.34	1	15.34	12.73	0.0118	
BC	3.03	1	3.03	2.51	0.1639	
A^2^	18.35	1	18.35	15.23	0.0080	
B2	0.5330	1	0.5330	0.4424	0.5307	
C2	542.82	1	542.82	450.49	< 0.0001	
Residual	7.23	6	1.20			
Lack of Fit	3.28	4	0.8212	0.4163	0.7936	Not significant
Pure Error	3.94	2	1.97			
Corrected Total	1450.68	15				

R^2^ = 0.9877, Predicted R^2^ = 0.9685, adjusted R^2^ = 0.9789, adequate precision = 35.64

The degree of crystallinity based on coding factors is shown in Eq 9.

$$\begin{array}{l} \text{Crystallinity}=77.88+2.55*\text{A}+1.26*\text{B}+11.66*\text{C}-0.1331*\text{AB}+1.38*\text{AC}+\\ 0.6*\text{BC}-0.9743*{\text{A}}^{2}-0.1660*{\text{B}}^{2}-7.26*{\text{C}}^{2} \end{array}$$ (9)

The degree of crystallinity based on real factors is shown in Eq 10.

$$\begin{array}{l} \text{Crystallinity}=-6.0107+0.6911*\text{Temp}+0.39045*\text{Time}+12.6954*\text{pH}-\\ 0.001479*\text{Temp}*\text{Time}+0.015385*\text{Temp}*\text{pH}+0.06837*\text{Time}*\text{pH}-\\ 0.001083*{\text{Temp}}^{2}-0.01845{\text{Time}}^{2}-0.80611{\text{pH}}^{2} \end{array}$$ (10)

In the final Eq., only factors with p ≤ 0.05 were retained, yielding a proposed model to predict the degree of crystallinity from the remaining system parameters is shown in Eq 11.

$$\begin{array}{l} \text{Crystallinity}=-5.8495+0.23976*\text{Temp}+0.41848*\text{Time}+13.08496*\text{pH}+\\ 0.015385*\text{Temp}*\text{pH}-0.001010*{\text{Temp}}^{2}-0.79964*{\text{pH}}^{2} \end{array}$$ (11)

### The effect of reaction parameters on degree of crystallinity response

#### Effect of hydrothermal temperature

**Fig 8:**
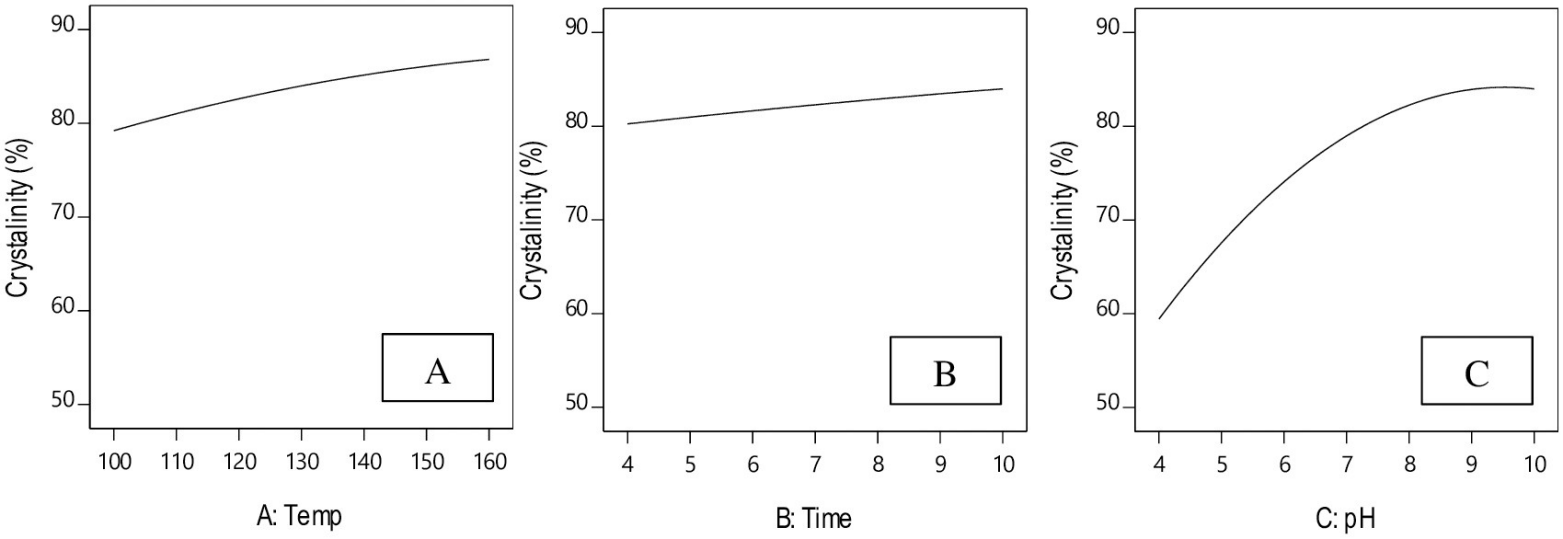
Effect of process parameters on degree of crystallinity. A: Hydrothermal temperature, B: hydrothermal reaction time, C: pH.

In Eq 11, the hydrothermal process temperature influences the degree of crystallinity and affects its response quadratically. Given the curves shown in Fig 8A, the degree of crystallinity increases with the temperature of the hydrothermal process. But according to Eq 11, the degree of crystallinity depends less on hydrothermal temperature and more on pH.

The research paper [32] studied the effect of temperature on the degree of crystallinity. With increased hydrothermal process temperature, the degree of crystallinity was found to increase. In another study [29], indicated that when temperature increases from 90°C to 200°C, crystallinity increases due to increased core growth.

#### Effect of hydrothermal process time

The effect of hydrothermal process time on the degree of crystallinity is shown in Fig 8B. With increased hydrothermal process time, the degree of crystallinity exhibited a first order increase, with ascending slope. In other words, the degree of crystallinity is directly related to the time of the hydrothermal process. When the hydrothermal process time was 4 hours, the degree of crystallinity was 82.53, which increased to 86.128 after 10 hours. In another study, [25] they evaluated the effects of hydrothermal reaction time (2 and 4 hours) on the degree of crystallinity and concluded that the degree of crystallinity increases with hydrothermal time.

#### Effect of pH

In Eq 11, the degree of crystallinity is most influenced by pH and hydrothermal reaction temperature. Based on the curves shown in Fig 8C, the degree of crystallinity increases with pH with a quadratic ascending slope. Therefore, the degree of crystallinity and pH are directly related, and the highest crystallinity nanosize crystals were formed at higher pH values [24].

**Fig 9:**
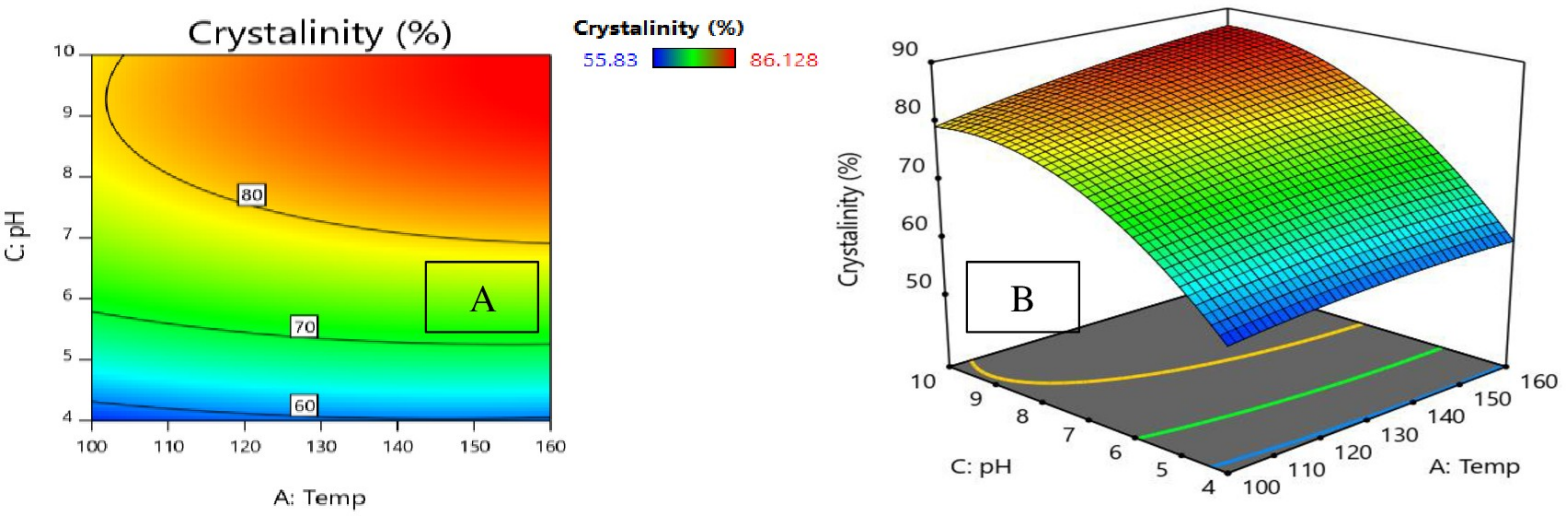
A: 2D contour plots and B: 3D surface plots for the effects of pH and temperature on the degree of crystallinity at time 10 h.

In our model of the degree of crystallinity expressed in Eq 11, pH interacts with hydrothermal temperature. One advantage of Surface Response Methodology over other methods is its ability to determine the interaction effect between parameters. When studying the interaction of two variables on the response of a model, 2D contour and 3D response level diagrams will be plotted. When plotting these curves, a constant has been assumed as a way of determining the simultaneous changes in the two other variables [33]. Hence, the 2D contour diagram (Fig 9A) and the 3D response surface (Fig 9B) for the degree of crystallinity have been plotted against pH and hydrothermal temperature. These diagrams (Fig 9) show that as pH increases, the degree of crystallinity remains low. In fact, when pH is at its maximum value (pH = 10), the degrees of crystallinity at the minimum (100°C) and maximum hydrothermal process temperatures (160°C) are 78.882 and 86.128, respectively. This indicates that hydrothermal process temperature has an effect on the degree of crystallinity.

**Fig 10:**
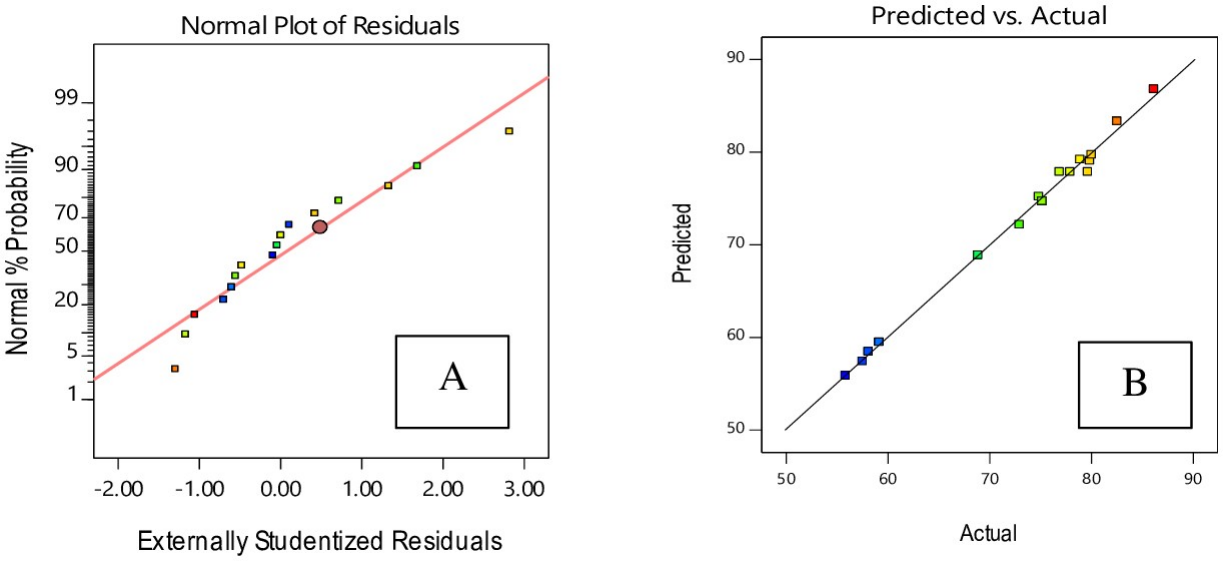
The residual plots for degree of crystallinity of HAp nano powder using hydrothermal method, based on CCD. A: normal probability; B: residuals versus predicted.

Fig 10A shows that residuals follow a normal distribution. The relation between the real data and predicted data is shown in Fig 10B. It may be seen that the created model is adequate because the residuals match the diagonal line [34].

**Fig 11:**
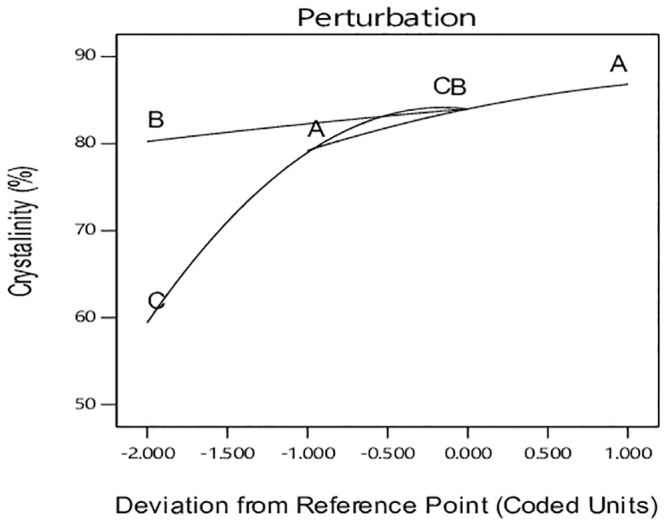
Perturbation chart for crystallinity response.

The perturbation plot in Fig 11 allows comparing all parameters at a special reference point in design space. The reference point is placed at the mid-point of all factors determined with zero code. The steep slope for pH indicates the sensitivity of response to this factor [16]. Hydrothermal reaction temperature has also steeper than hydrothermal reaction time and is more influential on the response degree of crystal size. However, this effect is weaker than that of pH. It should be noted that the effect of interactions is not observable in this diagram [16].

#### Improvement in crystal size

Based on the analysis of measurement responses, a polynomial model was selected by Design Expert 11. This software and the selected model are not aliased. A quadratic model was suggested by CCD Design for the same responses. Table 7 reports the results of variance analysis for crystal size based on L_002_ design, where the F-value is 93.55 and P-value less than 0.0001 indicates the validity of the model. In variance analysis, lower P-values and higher F-values indicate meaningful and acceptable data. In addition, Table 7 indicates other measures of adequacy: R^2^ adjusted, R^2^ predicted, and adequate precision ratio. Variance analysis found that pH and temperature and second order terms using these two parameters were meaningful. For these responses, the Step-Wise regression method was selected, and less meaningful terms with p ≥ 0.05 were automatically removed from the model [33].

**Table 7:** ANOVA table for crystal size, quadratic model.

Sorce	Sum of Squares	df	Mean square	F Value	P-value Prop>F	
Model	4883.74	9	542.64	93.55	< 0.0001	Significant
A-Temp	1524.56	1	1524.56	262.83	< 0.0001	
B-Time	114.93	1	114.93	19.81	0.0043	
C-pH	3113.93	1	3113.93	536.83	< 0.0001	
AB	2.07	1	2.07	0.3574	0.5718	
AC	72.16	1	72.16	12.44	0.0124	
BC	0.1030	1	0.1030	0.0178	0.8984	
A^2^	17.62	1	17.62	3.04	0.1320	
B2	19.33	1	19.33	3.33	0.1177	
C2	886.08	1	886.08	152.76	< 0.0001	
Residual	34.80	6	5.80			
Lack of Fit	32.57	4	8.14	7.29	0.1242	Not significant
Pure Error	2.23	2	1.12			
Corrected Total	4918.55	15				

R^2^ = 0.9702, Predicted R^2^ = 0.9246, R^2^adj = 0.9454, adequate precision = 32.69

The model for crystal size with terms based on coding factors is shown in Eq 12.

$$\begin{array}{l} Crystal\;size=45.40+9.00*A+2.68*B-18.84*C-0.5090*AB-3.00*AC-\\ 0.1135*BC-0.2218*A^{2}+1.03*B^{2}+9.45*C^{2} \end{array}$$ (12)

The model for crystal size with terms based on real factors is shown in Eq 13.

$$\begin{array}{l} Crystal\;size=79.4701+0.4299*Temp-1.34977*Time-16.5579*pH-\\ 0.0005656*Temp*Time-0.03371*Temp*pH-0.012607*Time*pH+\\ 0.000246*Temp^{2}+0.114009*Time^{2}+1.0503*pH^{2} \end{array}$$ (13)

The final mathematical model for L_002_ crystal size response and meaningful terms with p ≥ 0.05 based on real factors using Design Expert software is shown in Eq 14.

$$\begin{array}{l} \text{Crystal}\;\text{size}=70.2607+0.53367*\text{Temp}-0.56614*\text{Time}-16.52705*\text{pH}-\\ 0.033371*\text{Temp}*\text{pH}+0.10425*Time^{2}+1.04164*{\text{pH}}^{2} \end{array}$$ (14)

Eq 14 indicates factors with positive and negative effects on crystal size.

### The effect of process parameters on crystal size

#### Effect of hydrothermal temperature

**Fig 12:**
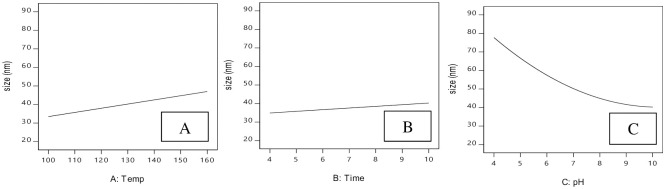
Effect of process parameter on crystal size. A: Hydrothermal temperature, B: hydrothermal reaction time, C: pH.

The effect of hydrothermal process temperature on nanoparticle size is shown in Fig 12A. As hydrothermal process temperature increases, nanoparticle size shows a first order increase with an ascending slope. In other words, the size of nanoparticles will increase with hydrothermal process temperature. At a hydrothermal process temperature of 100°C, nanoparticle size was 31.58 nm, and at 160°C, it reached 45.47 nm. Paper [25] studied the effects of the hydrothermal process on apatite nanocrystals characteristics. Their findings indicated that higher hydrothermal process temperature promotes crystal growth because it provides higher activity levels for smaller apatite crystals that are bound and grow along the C-axis. This increase in crystal size with temperature is in accordance with the results of other research [27].

#### Effect of hydrothermal reaction time

The effect of hydrothermal process time on the size of nanoparticles is shown in Fig 12B. As hydrothermal process time increases, nanoparticle size showed the first order increase with ascending slope. However, this size increase was less than that produced by pH and hydrothermal process temperature. At constant temperature and pH, when the time of hydrothermal process was 4 hours, the size of nanoparticles was 38.89 nm, but when time increased to 10 hours, nanoparticle size reached 45.47 nm.

Crystal growth intensifies with longer reaction times since the increased hydrothermal process time promotes solvent evaporation, oversaturating the remaining solution, which leads to crystal growth [25].

#### Effect of pH

**Fig 13:**
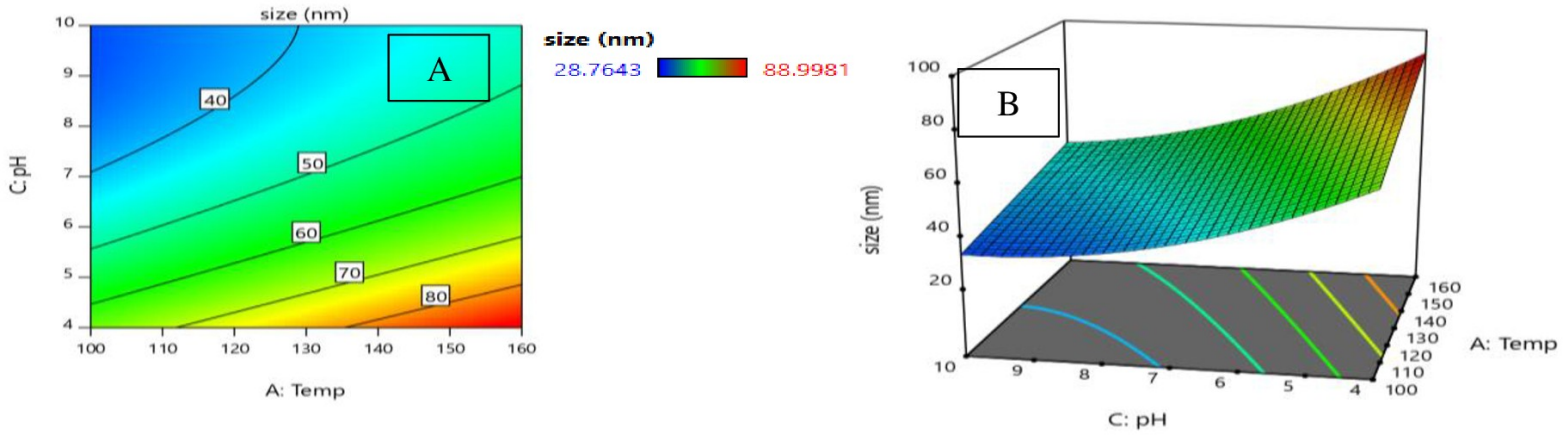
A: 2D contour plots and B: 3D surface plots for the effects of pH and temperature on the percent of crystal size at time 10 h.

According to Eq.14, the factor most influencing hydroxyapatite particle size is pH, which affects other particles quadratically and interacts with hydrothermal process temperature. As shown in Fig 12C, increasing pH reduces hydroxyapatite particle size with a quadratic descending slope. In other words, nanoparticle size has an inverse relation with pH. When pH = 4, particle size was 88.99 nm, whereas a pH value of 10 yielded nanoparticles of size 45.47 nm. The research [35] investigated the effect of pH on the size of hydroxyapatite crystals using a hydrothermal method and with eggshells as a calcium source. Their studies indicated that as the pH value increases from 5 to 9, the size of hydroxyapatite crystals also increases. In the model of particle size given in Eq 14, pH also influences hydrothermal process temperature. Therefore, a 2D contour diagram (Fig 13A) and 3D response surface (Fig 13B) of particle size against pH and hydrothermal process temperature are given. These diagrams show that increased hydrothermal temperature increases particle size. At a constant hydrothermal process time, nanoparticle size reduces with increased pH. Therefore, as indicated in the diagrams, when hydrothermal reaction time is constant, lower temperatures and higher pH yield nanoparticles with small sizes.

The study [18] synthesized crystalline and whiskered hydroxyapatite using a hydrothermal method. In that study, the effect of different temperatures (temperature = 60, 80, 100, 120, 140°C) and pH (pH = 6, 9, 14) on hydroxyapatite morphology were investigated. At a time of 24 hours, and with pH = 9 and temperature = 120°C, whiskered and bar form HAp with 8–20 nm diameter and length 40–600 nm was produced. This study indicates that pH value and temperature are significant parameters in altering the morphology and particle size.

**Fig 14:**
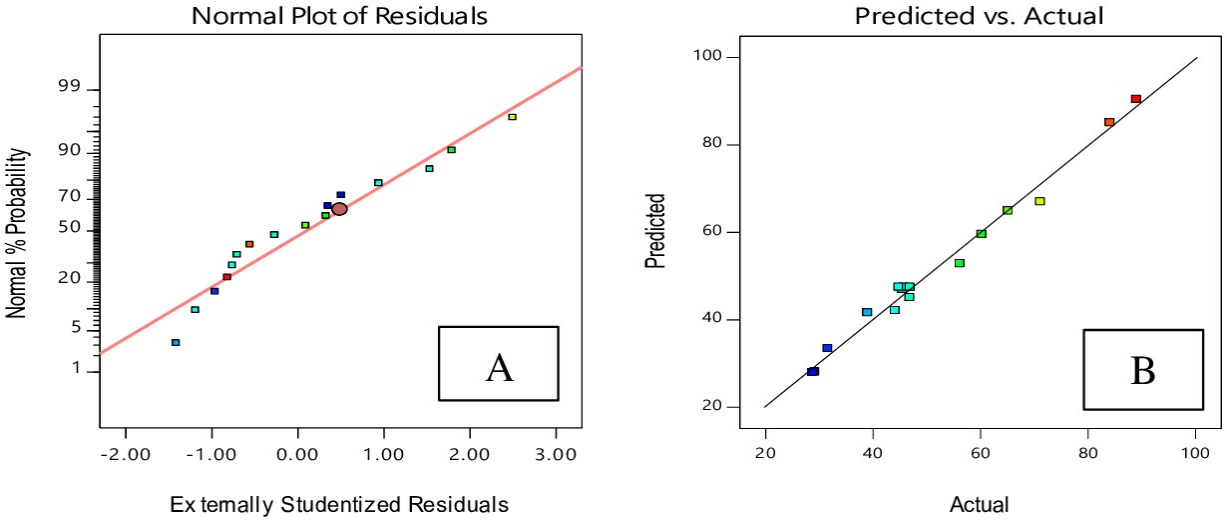
Residual plots for particle size of HAp nano powder, according to CCD. A: Normal probability; B: residuals versus predicted.

A normal probability versus residuals plot is shown in Fig 14A. From this Figure, the data match the diagonal line almost perfectly, indicating a normal distribution of errors and also that the model has a good fit. The relationship between actual and predicted data is shown in Fig 14B. From this Figure, it may be concluded that actual data and experimental data closely match. The correlation coefficient of R^2^ = 0.97 confirms a good fit.

**Fig 15:**
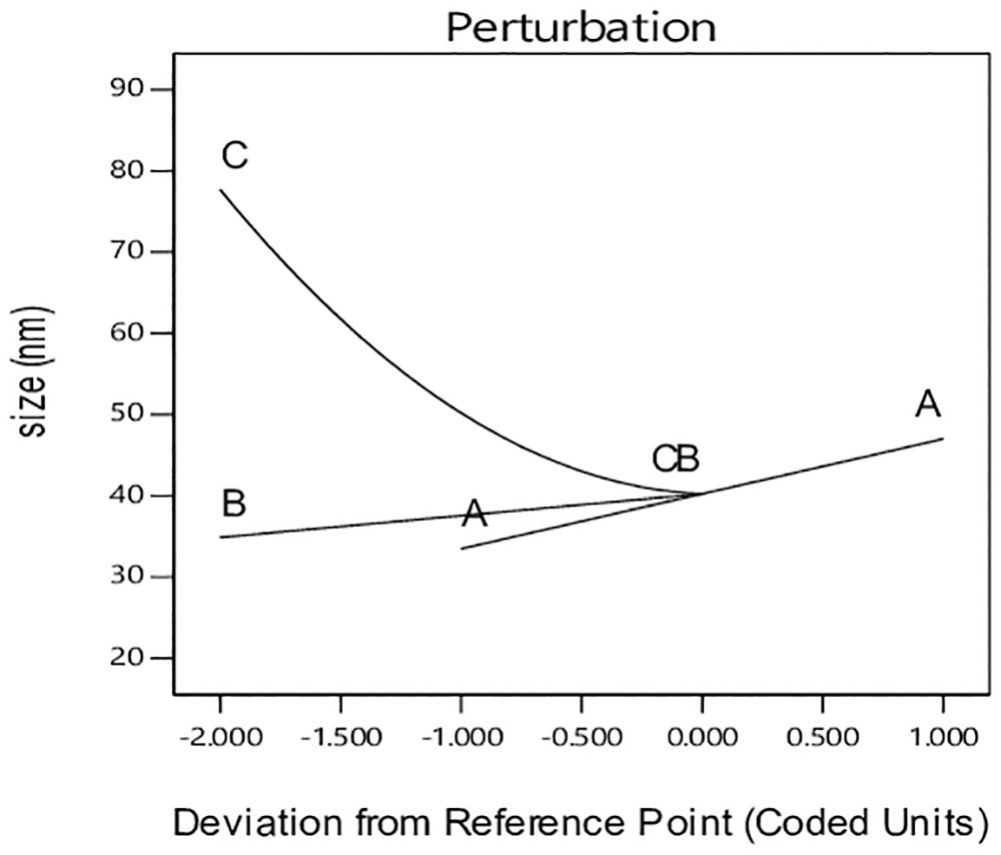
Perturbation chart for crystal size response.

Fig 15 shows the perturbation chart of the effect of process parameters on crystal size response. Based on Fig 15, a positive linear relation between reaction temperature and crystal size is seen, which is in accordance with the TEM results. The curve relation of the parameter indicates the lower effect on crystal size.

#### Optimization of HAp powder characteristics

**Fig 16:**
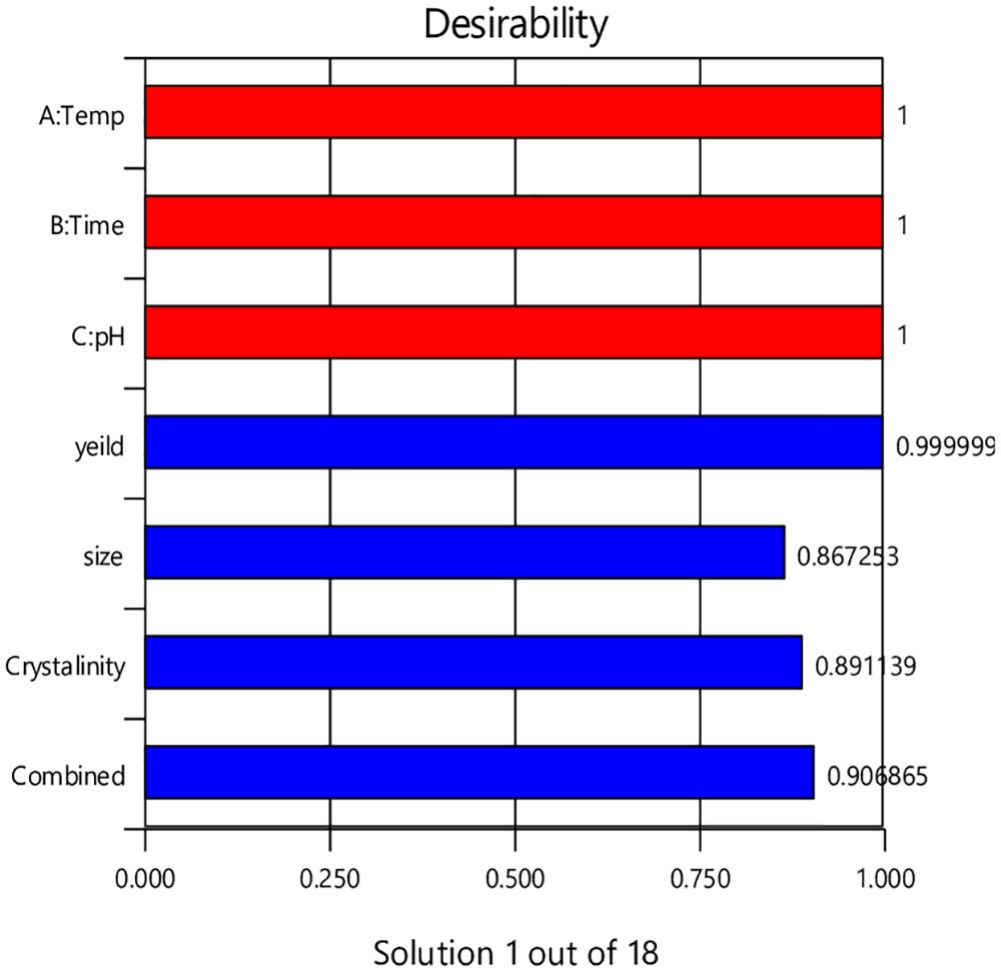
Desirability bar graph.

**Fig 17:**
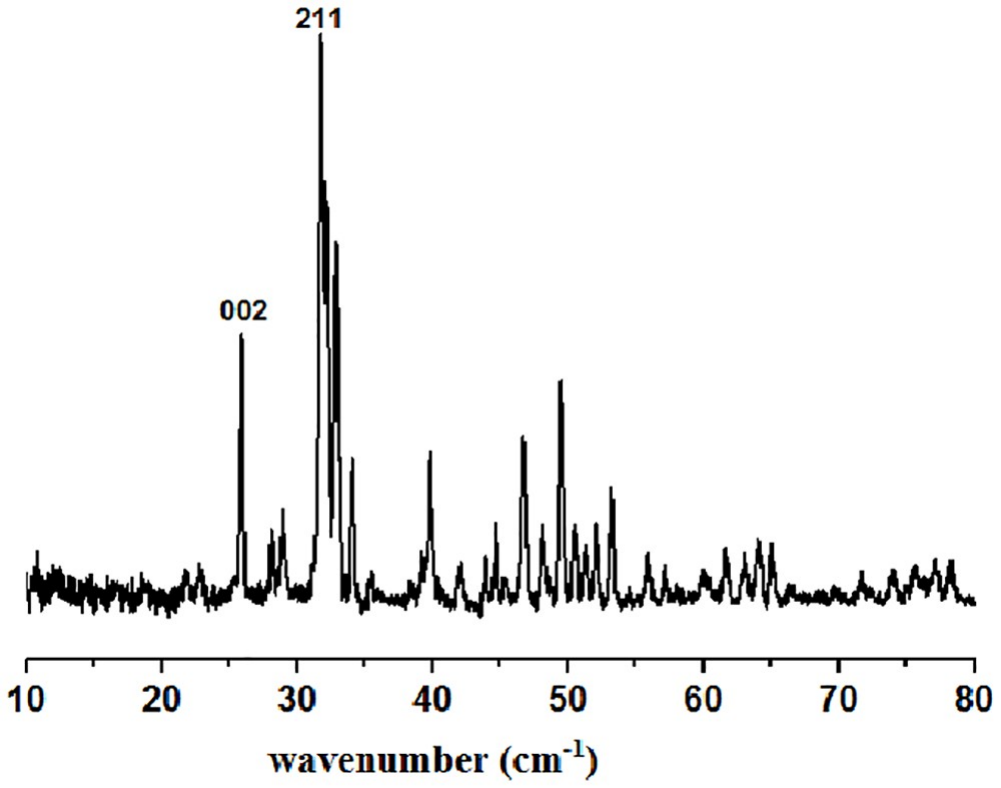
XRD pattern at optimum conditions (time = 10 h, temperature: = 130°C, pH = 10).

The characteristics of the synthesised HAp powder were optimized using Design Expert 11 software. The models developed earlier were used to enhance this process. Design Expert is capable of nominal and graphical optimization. Nominal optimization is done first because of its high efficiency in determining the desired area. This method can be used industrially to reduce the costs of controlling each factor (temperature, time, and pH) to achieve the desired response. Each response is given to optimize an aim and its importance level. The purpose of desirable optimization is to produce a product with higher purity and crystallinity, as well as the production of HAp powders at the nanoscale and with high yield. In order to synthesise hydroxyapatite powders with these desired characteristics, the degree of crystallinity and the yield of HAp powders should be maximized, and the size of the crystals in the L_002_ plane should be minimized (i.e. high yield, high crystallinity, and small size). This is to ensure the lowest level of impurity. The importance surface for each response was selected within the desired optimization. Yield and degree of crystallinity were given importance levels 3 and 4, respectively, whereas crystal size in the L_002_ plane was assigned importance level 5. The importance and goal of each optimized response are shown in Table 8. Design Expert is able to create possible solutions based on selected optimization indices. Desirability (0–1) has been shown for each solution. The preferred adjustments can be selected manually. The desirability bar graph for each parameter and response as well as the combined desirability by the CCD model is shown in Fig 16. From bar graph a value close to one is considered adept. The combined desirability with 0.90 shows with the solution (Temp 130°C, time 10 h and pH 10) can achieve 90% research goal (high yield and high degree of crystallinity and smallest size). The optimized HAp powder has characteristics to be used in SBF solution to evaluate the bioactivity of HAp powder using the hydrothermal method that can be produced by process parameters such as time and hydrothermal temperature and pH. Based on the experimental analysis (i.e. TEM, XRD, and FTIR) and response surface optimization, hydrothermal temperature = 130°C, pH = 10, and hydrothermal time = 10 h were selected as the optimal conditions. XRD results (Fig 17) indicated that the obtained HAp in optimal conditions is completely pure with a yield of 90%, degree of crystallinity of 83%, and crystal size of 38 nm. The high intensity and broad width of the peaks show that the particles are highly crystalline and nanoscale size, respectively (Fig 17). The results show the phase composition for the final HAp, as obtained through x-ray diffraction data. Based on Fig 17, there are no obvious peaks for the presence of other phases of calcium phosphate like β-TCP, α-TCP, and CaO; this indicates the purity of the prepared sample. XRD pattern of final HAp compared with ICCD standard curve of pure HAp (Fig 5A), which shows good agreement with similarity [36].

**Table 8:** Design optimization parameter.

Name	Goal	Lower Limit	Upper Limit	Importance
Yield	Minimize	46.18	91	3
Size	Maximize	28.7643	88.9981	5
Crystallinity	Minimize	55.83	86.128	4

**Fig 18:**
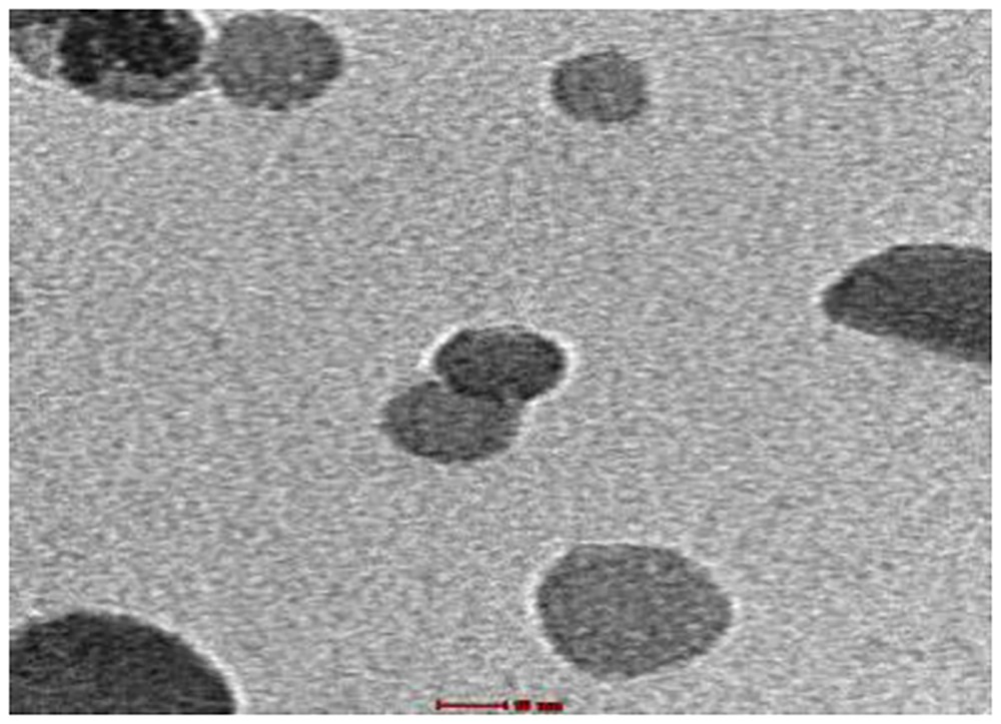
TEM result for HAp powder at optimal conditions (time = 10 h, pH = 10, temperature = 130°C).

**Fig 19:**
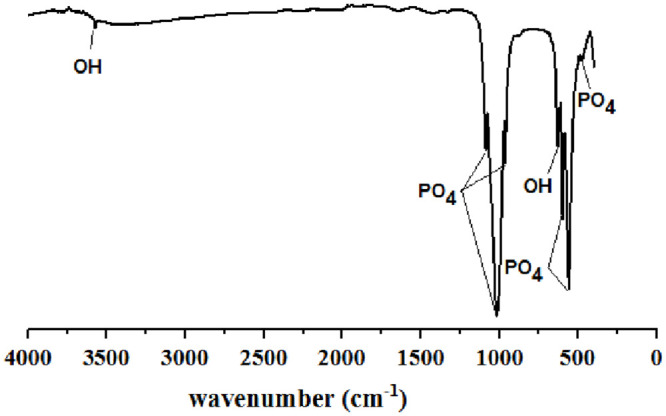
FTIR spectrum of HAp powder at optimal conditions (time = 10 h, pH = 10, temperature = 130°C).

TEM results (Fig 18) indicated that the obtained HAp particles are spherical with short bar shapes. Additionally, FTIR spectrum analysis (Fig 19) showed that the HAp obtained in optimal conditions has all the peaks of HAp. No other peaks in the FTIR spectra (Fig 19) clearly confirm the purity of the synthesized HAp nanostructures [37].

**Fig 20:**
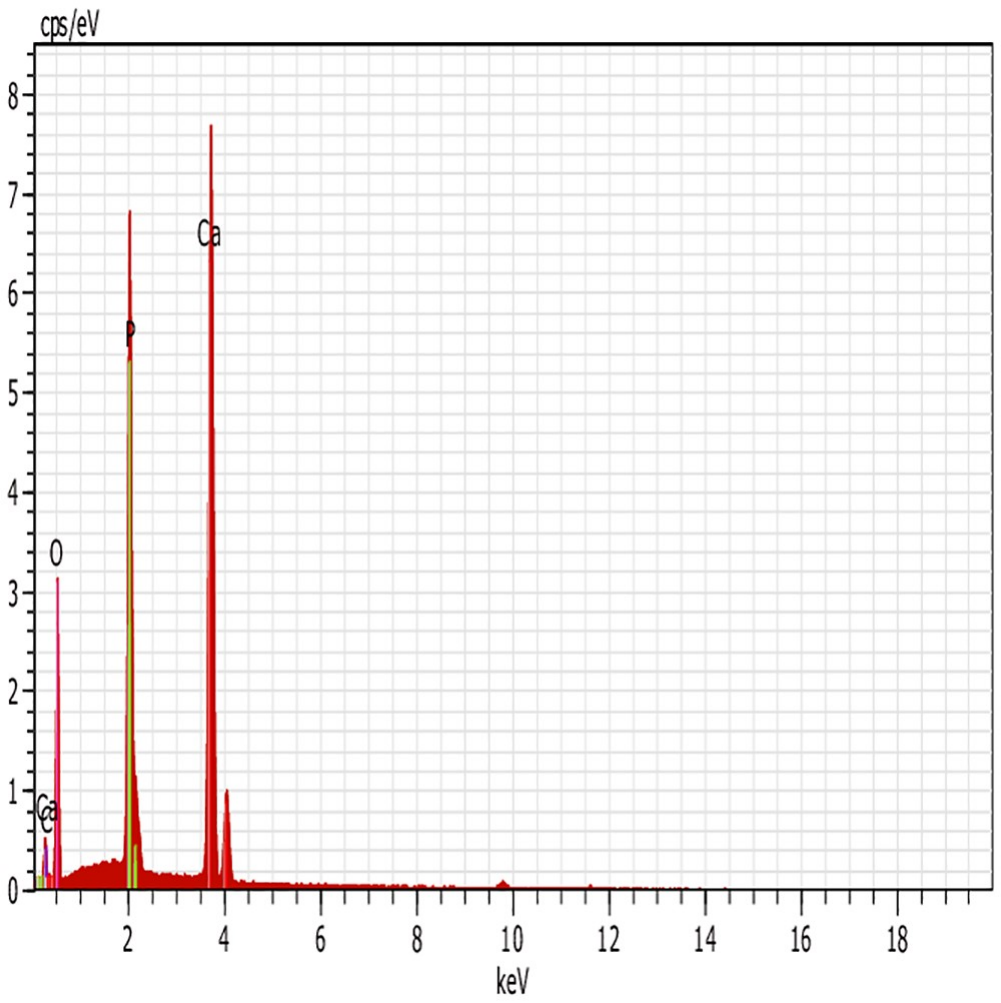
EDX result for HAp powder at optimal conditions (time = 10 h, pH = 10, temperature = 130°C).

The EDX spectrum analysis (Fig 20) shows the elemental composition of the final HAp nanopowder. The EDX result shows that the produced HAp nanopowder is composed of calcium and phosphorous with an oxygen amount. There are no significant phases observed apart from those elements, and the carbon peak is hardly seen in Fig 20. The absence of other elements attributed to the purity of the produced HAp nanopowder. The Ca/P ratio of final HAp is 1.676, which confirms the purity of produced HAp [37]. The XRD and FTIR results confirmed the EDX results, which show the high purity of produced HAp.

#### Bioactive behavior of HAp powder

**Fig 21:**
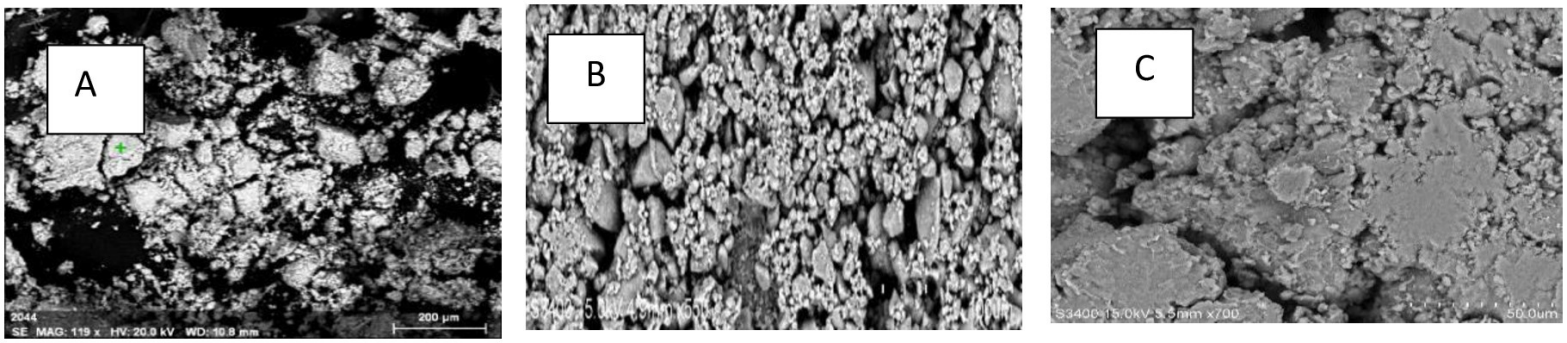
SEM images of HAp powder a) before and b) after immersion in SBF c) growth of apatite layer after immersion in SBF.

To evaluate bioactivity of the final HAp powder, immersion in Simulated Body Fluid (SBF) solution was carried out for 14 days under optimal conditions (temperature = 130°C, time = 10 h, pH = 10). The pH of the SBF solution and the morphology and compounds of its surface were studied. The nanometer hydroxyapatite sample caused changes in the pH of the solution, indicating the presence of ions in the solution from this material. The SBF solution had an initial pH of 7.4. After 14 days of nanometer hydroxyapatite immersion in the SBF solution, the solution pH decreased to about 7. (i.e. HAp dissolved in SBF leads to decreasing pH of SBF). [37]. Fig 21 shows the SEM images of HAp powder before (Fig 21A) and after immersion (Fig 21B) in SBF. These results indicate that after immersion of the samples in SBF, some layers of biological apatite were formed. Since the hydroxyapatite has a nanometer dimension, it has a high surface area, so its contact with the adjacent solution is greater. Therefore, the transfer of ions from the surface of the material into the SBF is faster than the hydroxyapatite with micron dimensions [37]. Since surface HAp has functional groups with a negative charge (hydroxyl and phosphate groups), calcium with a positive charge inside the SBF is absorbed by hydroxyl and phosphate ions on surface HAp, giving it a positive charge. This positive charge is absorbed by the negative charge present in the SBF solution. Therefore, an apatite layer is formed on the HAp surface (Fig 21B) [38]. These findings agree with the results found by previous researchers [38–40]. Xingdong, Pin [39] have reported biological layer formation of apatite on HAp powder surface in a simulated solution.

**Fig 22:**
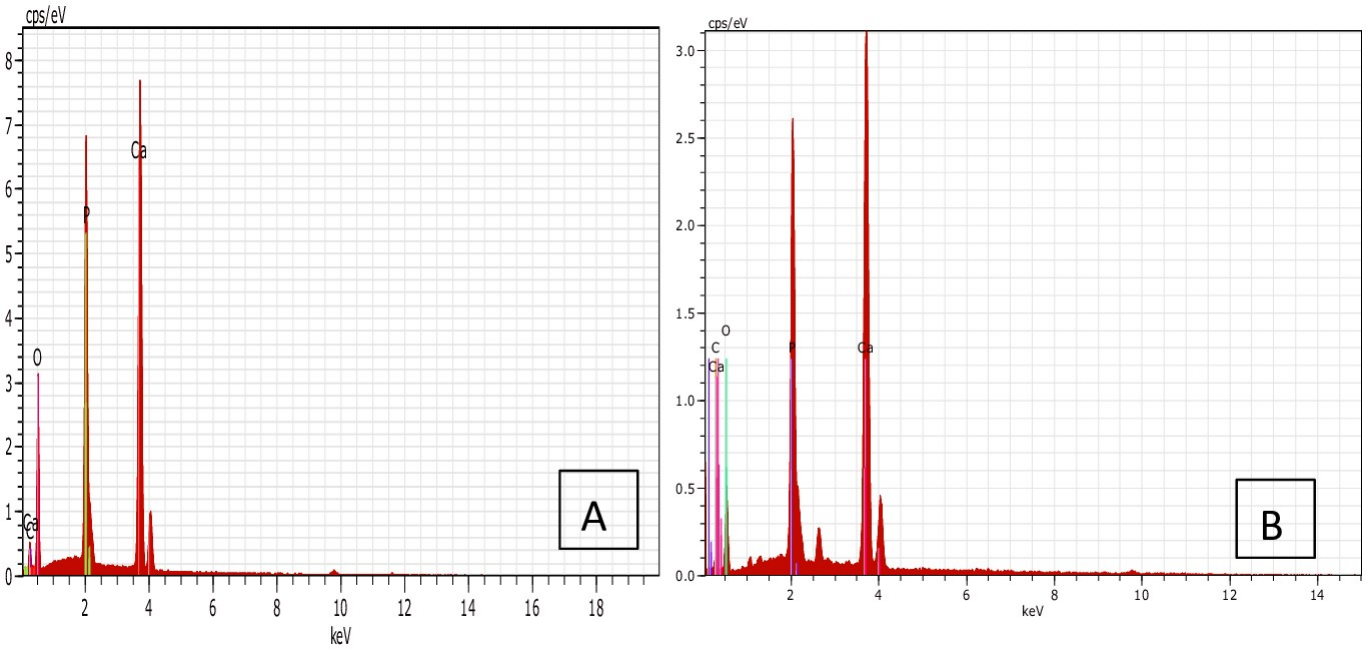
EDX spectrum of HAP. a) before, and b) after immersion in SBF.

On the other hand, HAp nanocrystals are regarded highly due to their improved biological performance, including bone tissue connection and the formation of new bone on their surface. The formation of a porous apatite layer on the HAp surface in SBF solution is shown in Fig 21C, which indicates suitable level bioactivity of produced HAp [40]. Furthermore, the results of bioactivity evaluation studies are based on EDX analysis. Fig 22 shows the EDX result and Ca, P, and O elements before (Fig 22A) and after (Fig 22B) immersion in the SBF solution. Due to the formation of an apatite layer on the HAp surface after immersion in SBF solution, the peaks belonging to Ca and P have increased.

## Conclusion

Hydroxyapatite nanopowder was successfully synthesized using a hydrothermal method using (Ca (NO_3_)_2_.4H_2_O) and ((NH_4_)_2_HPO_4_) as sources of calcium and phosphate, respectively. The importance of process parameters (time, hydrothermal temperature, and pH) was evaluated using RSM and CCD design. A quadratic model was selected as significant by both RSM methodology and CCD design. It may be concluded that pH is the most influential variable on the yield, crystallinity, and crystal size of hydroxyapatite nanopowder. The optimum process parameters were obtained to be pH = 10, temperature = 130°C and time = 10 h. This study showed that HAp with high crystallinity and nanoscale has high bioactivity in SBF solution. The formation of a porous apatite layer on the HAp surface in SBF indicates a suitable level of bioactivity.

## Supporting information

S1 Data (RAR)
